# Blunted Response to Combination Antiretroviral Therapy in HIV Elite Controllers: An International HIV Controller Collaboration

**DOI:** 10.1371/journal.pone.0085516

**Published:** 2014-01-17

**Authors:** Faroudy Boufassa, Jérome Lechenadec, Laurence Meyer, Dominique Costagliola, Peter W. Hunt, Florencia Pereyra, Steve Deeks, Gianfranco Pancino, Olivier Taulera, Mathias Lichterfeld, Pierre Delobel, Asier Saez-Cirion, Olivier Lambotte, for the ANRS CO18 HIV Controllers Cohort, the Cascade Collaboration in Eurocoord, the SCOPE Cohort and the International HIV Controllers Study

**Affiliations:** 1 Inserm, CESP Centre for research in Epidemiology and Population Health, Epidemiology of HIV and STI Team, le Kremlin-Bicêtre, France; 2 Univ Paris-Sud, Le Kremlin Bicêtre, France; 3 AP-HP, Service de Santé Publique, Hôpital de Bicêtre, le Kremlin Bicêtre, France; 4 UPMC Univ Paris 06, Paris, France; 5 INSERM, Paris, France; 6 Laboratory Medicine, Departments of Medicine, Epidemiology, and Biostatistics, University of California San Francisco, San Francisco, California, United States of America; 7 The Ragon Institute of Massachusetts General Hospital, Massachusetts Institute of Technology, and Harvard University, Cambridge, Massachusetts, United States of America; 8 Hôpital Saint-Louis, Paris, France; 9 Infectious Disease Division, Massachusetts General Hospital, Boston, Massachusetts, United States of America; 10 Service des Maladies Infectieuses et Tropicales, Hôpital Purpan, Toulouse, France; 11 INSERM, Toulouse, France; 12 Institut Pasteur, Unité de Régulation des Infections Rétrovirales, Paris, France; 13 AP-HP, Service de Médecine Interne, Hôpital de Bicêtre, Le Kremlin-Bicêtre, France; Infectious Disease Service, United States of America

## Abstract

**Objective:**

HIV “elite controllers” (ECs) spontaneously control viral load, but some eventually require combination antiretroviral treatment (cART), due to a loss of viral control or a decline in CD4 T-cell counts. Here we studied the CD4 T-cell count dynamics after cART initiation among 34 ECs followed in U.S. and European cohorts, by comparison with chronically viremic patients (VIRs).

**Methods:**

ECs were defined as patients with at least ≥5 viral load (VL) measurements below 400 copies/mL during at least a 5-year period despite never receiving ART and were selected from the French ANRS CO18 cohort, the U.S. SCOPE cohort, the International HIV Controllers study and the European CASCADE collaboration. VIRs were selected from the ANRS COPANA cohort of recently-diagnosed (<1 year) ART-naïve HIV-1-infected adults. CD4 T-cell count dynamics after cART initiation in both groups were modelled with piecewise mixed linear models.

**Results:**

After cART initiation, CD4 T-cell counts showed a biphasic rise in VIRs with: an initial rapid increase during the first 3 months (+0.63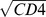/month), followed by +0.19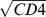/month. This first rapid phase was not observed in ECs, in whom the CD4Tc count increased steadily, at a rate similar to that of the second phase observed in VIRs. After cART initiation at a CD4 T-cell count of 300/mm^3^, the estimated mean CD4 T-cell gain during the first 12 months was 139/mm^3^ in VIRs and 80/mm^3^ in ECs (p = 0.048).

**Conclusions:**

cART increases CD4 T-cell counts in elite controllers, albeit less markedly than in other patients.

## Introduction

Current guidelines in industrialized countries shift to recommend antiretroviral treatment for all HIV-infected patients. Combination antiretroviral therapy (cART), whether started during the primary infection or in the chronic phase of HIV-1 infection, generally leads to a biphasic increase in CD4 T cell counts, with a rapid increase during the first months, followed by a more gradual rise [1]–[4]. At least 80% of patients are expected to achieve viral control after 6 months of treatment, with a median CD4 T cell increase of about 100 CD4/mm^3^ [5]–[7].

“Elite Controllers” (ECs) are HIV-1-infected individuals in whom viral replication is spontaneously controlled for several years [8]–[12]. ECs represent about 1% of all HIV-1-infected patients [13]–[15]. The time course of CD4 T cell counts is variable in ECs and eventual loss of HIV control or marked CD4 T cell depletion sometimes necessitates cART initiation. Chronic immune activation and inflammation are another possible indication for cART in these patients. Two previous studies have examined the impact of cART in ECs. In the first, the CD4 T cell increase in the six ECs studied tended to be smaller than in other patients [16]. The second [17], more recent study, examined the impact of cART on immune activation in 16 ECs: markers of T cell activation and HIV RNA load decreased during 24 weeks of cART, but no change in the CD4 T cell count was noted. Additional information is therefore necessary to clarify the impact of cART on the CD4+ T cell count in ECs.

Here, we studied the response to first-line cART among 34 ECs participating in several HIV controller cohorts in the USA and Europe. CD4 T cell count dynamics were compared to those observed in chronically infected patients also starting first-line cART at the chronic stage.

## Methods

ECs were identified from the French ANRS CO18 HIV Controllers cohort [18], the San Francisco Bay Area cohort (SCOPE Cohort) [19], the International HIV Controllers Study [20], and the CASCADE collaboration which includes HIV seroconverters from 28 European, Canadian, Australian and sub-Saharan African cohorts [21]. In order to harmonize the definition of ECs across the cohorts, we adopted the ANRS definition [21], i.e. patients with a minimum of 5 viral load (VL) measurements below 400 copies/mL over a minimum of 5 years despite never receiving ART. Interestingly, all the selected patients also fulfilled the American elite controller definition, i.e. at least 3 VL determinations below the assay detection limit (generally <50 copies/mL) spanning greater than 12 months in the absence of ART for ≥1 year before and during the period of virologic control [12], [22]. We studied ECs who received cART for whatever reason (VL increase or CD4T cell loss), apart from transient prevention of mother-to-child transmission, and who had at least two available CD4 cell counts after cART initiation, in order to model the dynamics of CD4 T cell counts.

VIRs were selected from the ANRS COPANA Cohort in which recently diagnosed (<1 year) cART-naïve HIV-1-infected patients have been enrolled since 2004. Among 800 enrolled individuals, we studied the 478 patients who initiated cART and who had at least two CD4 cell counts after cART initiation. All the participants gave their written informed consent. Clinical investigations were conducted according to the principles of the Declaration of Helsinki and all the cohorts were approved by local ethics committees. Ethical approvals for each cohort are detailed in the appendix.

### Statistical Analysis

Baseline demographic and immuno-virological characteristics at the time of cART initiation were described by the median and interquartile range for continuous variables, and percentages for discrete variables. CD4 T cell counts and HIV viral loads at cART initiation were defined as the last available values before treatment initiation. Univariate and multivariate (adjusted for sex and age at cART initiation) mixed-effect linear models in ECs and VIRs patients were used to estimate changes in CD4 cell counts from cART initiation. These models were chosen in order to take into account the fact that the subjects had repeated CD4 measurements. The best models were chosen by applying likelihood ratio tests or Akaike information criterion. Given the available number of CD4 T cell counts, we chose to model CD4 T cells dynamics for the first 15 months after cART initiation, during which the median numbers of available CD4 measurements was 4 [IQR: 3–6]. Square root transformation of CD4 T cell counts was used to fulfill the model assumptions. To facilitate understanding of the results, estimates were also provided in absolute CD4 T cell numbers by squaring intercept and increase given by the model. SAS (Version 9.3, 2003; SAS Institute Inc., Cary, NC, USA) and STATA software (Version 12.0, 2009; Stata Corp., College Station, Texas) were used for statistical analysis.

## Results

### Characteristics of ECs and VIRs

Main characteristics of ECs and VIRS are resumed in Table 1. We did not observe any difference in gender between groups, but men who have sex with men were less frequent among ECs than among VIRs (32% vs. 62% respectively; p<0.001). More ECs were IVDUs or infected through other haematogenous routes than VIRs (41% vs 2%; p<0.001), although some data were missing for this parameter. Patients of sub-Saharan African, Caribbean and African-American origin were less frequent among ECs than among VIRs (11.8% vs 40.6%; p<0.001). As expected, the year of infection was less recent in ECs than in VIRs, consequently, the time from HIV diagnosis to ART initiation was much longer in ECs (median 16 years vs 1 year; p<0.001). Finally, ECs were younger than VIRs at HIV diagnosis but older at cART initiation (both p<0.001).

**Table 1:** Characteristics at baseline and at cART initiation in 34 elite Controllers (ECs) and 478 chronically viremic (VIRs) patients from the ANRS COPANA Cohort.

Characteristics	Elite Controllers	COPANA Cohort Study	p
**Patient recruitment (TreatedECs/TotalECS** * **)**			
ANRS CO18 cohort	9/186		
CASCADE collaboration	12/140		
SCOPE cohort	7/100		
The International HIV Controllers Study	6/450		
Women (%)	9 (26.5)	149 (31.2)	0.57
**Route of infection**			
Sexual	19 (55.9)	409 (85.6)	<0.001
IVDU	10 (29.4)	3 (0.6)	
Other hematogenous routes (hemophiliacs or transfusions)	3 (8.8)	6 (1.3)	
Unknown	2 (5.9)	60 (12.6)	
**Sexual preference among men**	n = 25	n = 329	
Heterosexual	9 (36.0)	123 (37.4)	<0.001
Men who have sex with men	8 (32.0)	204 (62.0)	
Unknown	8 (32.0)	2 (0.6)	
**Ethnicity**			
White	19 (55.9)	270 (57.1)	<0.001
BSSA/Car/BA**	4 (11.8)	192 (40.6)	
Other	3 (8.8)	11 (2.3)	
Unknown	8 (23.5)	–	
Year of HIV diagnosis	1992 [1986–1999]	2005 [2004–2006]	<0.001
Age at HIV diagnosis	29 [25]–[33]	35 [29–44]	<0.001
**HCV and HBV serostatus**			
Both positive	5 (15.0)	14 (3.0)	
HCV^+^ and HBV^−^	5 (15.0)	8 (2.0)	
HCV^−^ and HBV^+^	1 (3.0)	271 (57.0)	
Both negative	17 (50.0)	141 (29.0)	
At least one of the two	5 (15.0)	33 (7.0)	
Unknown	1 (3.0)	11 (2.0)	
AIDS diagnosis before cART initiation	2 (6.0)	55 (12.0)	0.41
Year of cART initiation	2008 [20006–2010]	2007 [2005–2008 )	0.002
Age at cART initiation	45 [38–51]	37 [30–45]	0.0001
Time frome HIV-1 diagnosis to cART initiation (years)	16 [9]–[20]	1 [0–1]	<0.001
**Initial cART regimen**			
NRTI alone	2 (5.9)	17 (3.6)	
NRTI+Integrase Inhibitors	5 (14.7)	–	
NRTI+PI	9 (26.5)	307 (64.2)	
NRTI+NNRTI	15 (44.1)	137 (28.7)	
Others	3 (8.8)	17 (3.5)	
Median CD4/mm^3^ at cART initiation	268 [212–445]	255 [161–327]	
Mean CD4/mm^3^ at cART initiation (range)	348 (95–1249)	257 (2–998)	0.02
Median viral load log_10_ copies/mL at cART initiation	2.0 [1.7–2.6]	4.8 [4.2–5.3]	
Mean viral load log_10_ copies/mL at cART initiation (range)	2.2 (1.3–3.4)	4.7 (1.6–6.5)	<0.001

Data are median [IQR] or n (%);

According to own cohort’s definitions of ECs;

SSA/Car/BA = Sub-Saharan African, Caribbean or African-American origin.

Fifty percent of ECs were HCV and HBV seronegative while compared to only 29% of VIRs, while 15% of ECs were seropositive for both HCV and HBV compared to only 3% of VIRs. The proportion of HCV positive/HBV negative patents was higher among ECs than among VIRs patients (15% vs 2%) while the proportion of HCV negative/HBV positive was higher in VIRs patients (3% vs 57%). This latter difference was consistent with the higher frequency of sub-Saharan African, Caribbean and African-American origin among VIRs and the higher rate of HCV positivity among ECs was consistent with their higher frequency of infection through IVDU or other haematogenous route of infection. As expected, AIDS diagnosis before cART initiation was very rare in ECs with only two cases (low-grade lymphoma and Pulmonary Tuberculosis) vs 55 (12%) cases in VIRs patients (27.3% Pneumocystosis Pneumonia (PCP) and 20.0% Pulmonary Tuberculosis).

The initial cART regimen in ECs mainly consisted of NRTI+NNRTI (44.1%, mostly 2 NRTI+1 NNRTI) followed by NRTI+PI in 26.5% of cases; 14.7% of ECs received a combination of NRTI+integrase inhibitors. In contrast, 64.2% of VIRs received NRTI+PI and 28.7% received 2 NRTI+1 NNRTI.

The respective median CD4 cell counts in the ECs and VIRs, before treatment initiation, were 268/mm^3^ (range: 95–1249/mm^3^) and 255/mm^3^ (range: 2–998/mm^3^) (Wilcoxon rank sum test: p = 0.02) and the respective median viral loads were 2.0 log copies/mL (range: 1.3–3.4 copies/mL) and 4.8 log copies/mL (range: 1.6–6.5/copies/mL) (p<0.001).

cART initiation in ECs appears to have been prompted more by a decline in CD4 T cells than by a loss of viral control. In ECs, baseline viral load was measured a median of 38 days [IQR:−73; 0 day] before cART initiation: viral load was <50 copies/mL in 38% (n = 13) of patients and <400 copies/mL in 79%, and >1000 copies/mL in 18%. The median CD4 cell count in ECs was low with a wide range of individual values (95 to 1249/mm^3^).

### CD4 Cell Count Kinetics in ECs after cART Initiation

In ECs the first on-treatment CD4 cell T count was measured after a median of 27.4 days [3.0–42.6 days]. Several piecewise models were tested. The best fit was obtained with a model having only one slope of CD4 T cells. The mean intercept was estimated to be 17.79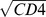 [16.04–19.53]. The mean CD4 T cell increase was estimated to be +0.19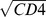 [+0.06-+0.32] per month until month 15, a value significantly different from zero (p = 0.006). For example, an EC who started cART at 316 CD4/mm^3^ (i.e. squared intercept) would reach counts of 337, 358 and 402 CD4/mm^3^, respectively, 3, 6 and 12 months after cART initiation. Introduction of sex and age at cART initiation in the model had no marked impact on these results. The CD4 cell count at cART initiation, categorized as <350 (n = 20) and ≥350 CD4/mm^3^ (n = 14) did not influence the CD4 cell slope, and neither did VL categorized as <50 vs ≥50 copies/mL. To note, the slope of the CD4 T cells in the 6 patients who initiated cART with a viral load >1000 copies/mL was not much steeper (+0.17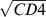 per month) than the mean slope estimated in other ECs. Despite the small sample of HCV^neg^ patients (n = 11), the estimated slope of the CD4 T cells showed, as in other ECs, a monophasic phase of increase after cART initiation.

### Differences in On-treatment CD4 T Cell Kinetics between ECs and VIRs

We then compared these CD4 T cell kinetics with those observed in VIRs from the ANRS COPANA cohort. Among VIRs, the median CD4 cell count at cART initiation was 255/mm^3^ [range: 2–998] and median viral load was 4.7 log copies/mL [range: 1.6–6.5]. The on-treatment CD4 T-cell count was measured a median of 27 days after cART initiation and the median number of CD4 T-cell counts obtained during the first 15 months of treatment was 5 [IQR:4–7]. Several piecewise models (one or more slopes, nodes at different times after cART initiation) were tested for VIRs. The best fit was obtained with a two-slope model with a node at 3 months following cART initiation, in agreement with previous reports [1]–[4]. According to this model, the mean intercept was estimated at 17.03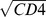 [16.52–17.55]. A marked initial increase of +0.63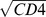 [+0.50–+0.77] per month was observed during the first 3 months, followed by a significant but more gradual increase of +0.19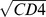 [−0.09−+0.48] per month (both <0.001). For example, a VIR starting cART at 290 CD4/mm^3^ (i.e. squared intercept) would reach 358 CD4/mm^3^ at month 3 (average +23 CD4 cells/month) and 427 CD4/mm^3^ (average +8 CD4 cells/month from month 4).

**Figure 1:**
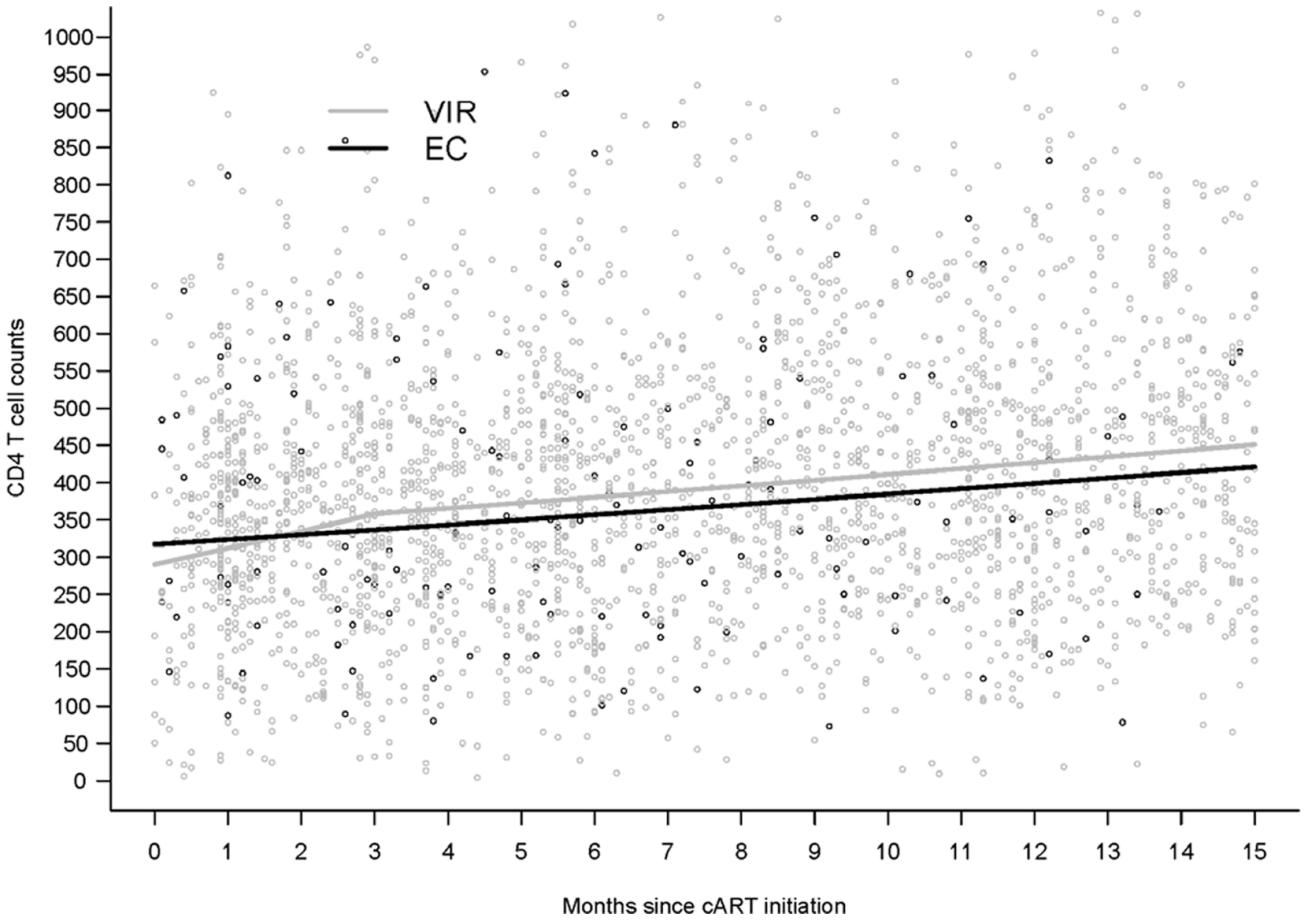
Estimated CD4 cell count dynamics in viremic patients (VIRs: n = 478) and elite controllers (ECs: n = 34) in mixed-effect linear models (mean CD4 T cells gain after 12 months of cART was significantly lower in ECs than in VIRs (p = 0.048)).

Figure 1 describes CD4 T cell dynamics during the first 15 months of cART in each group. By including data from the two groups in the same model, we were able to compare the CD4 gain. Twelve months after treatment initiation, the mean CD4 T cell gain was significantly larger in the VIRs than in ECs (p = 0.048) whatever the baseline count. Adjustment for sex and age at cART initiation did not affect these results. For an EC and a VIR beginning cART at a similar CD4 cell count of 300/mm^3^, the respective mean gain at 12 months would be 80/mm^3^ and 139/mm^3^.

The median year of cART initiation was 2008 in both groups, but the initial cART regimens differed significantly between ECs and VIRs (p<0.001): an NNRTI-based combination was more frequently prescribed to ECs than VIRs, while a PI-based combination was more often prescribed to VIRs than ECs. Only ECs received NRTI+integrase inhibitor (raltegravir^©^) combination. The number of ECs was too small to examine the influence of the different cART regimens. However, NNRTI-based cART was associated with a two-phase CD4 T-cell increment in VIRs, indicating that the absence of the first slope in ECs was unlikely to be due to a different choice of cART regimens.

In summary, the CD4 T-cell increment in chronically viremic patients starting first-line cART was biphasic with an initial rapid increase during the first 3 months followed by a more gradual increase of +0.19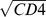 per month. ECs experienced no such initial rapid increment and their monophasic CD4 T-cell slope was very similar to the second slope observed in VIRs. As a result, the mean CD4 T-cell gain 12 months after cART initiation was significantly smaller in ECs than in VIRs.

## Discussion

The main result of this study is that cART increases the CD4 T-cell count in elite controllers, albeit relatively slowly, even in ECs who had experienced a large decline in their CD4 T cell count during the period of spontaneous control (50% of the ECs had counts below 268/mm^3^ at cART initiation). Two previous studies have studied the response to cART in ECs. The first was too small to draw firm conclusions [16], while the second showed no significant increase in the CD4 T cell count [17]. However, patients in the latter study had a higher median CD4 T cell count (616/mm^3^) than those in our study (268/mm^3^). Moreover, the patients in our study were treated for much longer than those described by Hatano et al. (15 vs 6 months).

Results from our study strongly suggest that the low levels of viral replication detectable with ultra-sensitive plasma HIV RNA assays in nearly all ECs [23] contribute to immunological attrition. Interestingly, viral escape seems to be both a rare event and a rare indication for cART in ECs, as only 6 patients in our study started cART at VL>1000 copies/mL. Another major finding of our study is that CD4 T-cell recovery is far less rapid and marked in ECs than in chronically viremic patients also starting a first-line cART. There are several possible explanations for this difference. First, high viral load at cART initiation, in chronically viremic patients, is associated with a steep initial increase in CD4 T-cell numbers, whereas the ECs in our study had undetectable or low viral load values at treatment initiation [6], [7], [16]. Second, although the seroconversion dates were not known for most of the study-patients, time since diagnosis was significantly longer in ECs than in VIRs. It is possible that durable T-cell-mediated control of infection, which would require continuous renewal of effective HIV-specific CD4 and CD8 T cells, may cause an exhaustion of hematopoietic progenitor cells in some ECs. Along these lines, severe CD4 T cell loss in ECs has been linked with impaired lymphopoiesis [24] and reduced thymic output [25]. Third, hepatitis C virus co-infection was more frequent in ECs than in VIRs in our study (32.3% vs 5.0%) [26]. HCV co-infection has been associated with a lower recovery of CD4 T cell counts during antiretroviral therapy [27]–[28], but conflicting results exist on this regard [29]. Limited redistribution of CD4 T cells from lymphoid tissues might also explain the lack of an initial, rapid CD4 cell increment in ECs, as this is the main mechanism underlying the first phase of CD4 T cells recovery in VIRs starting cART [30]. Finally, high levels of systemic inflammation correlate with low CD4 T cells counts in ECs [31] and are attenuated by cART [17].

This relatively slow CD4 cells recovery implies that it would take much longer for an elite controller to reach a target count of around 500/mm^3^. From a clinical point of view, early cART initiation is important for all patients with a sustained decline in their CD4 T cell count in order to avoid lengthy periods with persistently low CD4 T cell counts during treatment. It is important to keep or recover an adequate CD4 T cell count as AIDS-related morbidity in HIV-infected patients increase significantly at counts below 750/mm^3^ [32].

One limitation of this study is that follow-up on cART (15 months) was too short to determine long term immunological outcome. Moreover, the study population was too small to explore whether particular antiretroviral combinations may be more effective than others in ECs. CD4 T-cell percentages were also unavailable for most of our patients and we could not therefore compare patients with a decrease in both the CD4 T-cell count and percentage with those who became lymphopenic but had a stable percentage of CD4 T cells. Finally, it remains to be seen whether elite controllers are still able to control viral replication spontaneously after cART interruption and CD4 T cell recovery [33], but personal unpublished observational data from pregnant women in our cohorts suggest that this could be the case.

This study provides important information for clinicians caring for HIV elite controllers. Loss of CD4 T cells below 750/mm^3^ is associated with AIDS-defining events in other patients [32]. The best ART combination for these patients remains to be defined. An interesting therapeutic option could be an addition of immuno-modulatory drugs to cART in elite controller.

## Supporting Information

Appendix S1 (DOC)
