# Supplementary material for: Blunted Response to Combination Antiretroviral Therapy in HIV Elite Controllers: An International HIV Controller Collaboration
Source: PLoS One. 2014 Jan 17;9(1):e85516. doi: 10.1371/journal.pone.0085516 (PMC3894966; doi:10.1371/journal.pone.0085516)
Supplement: Appendix S1 — (DOC) [file pone.0085516.s001.doc]

**Appendix:**

**Ethical approvals by cohorts**

For the **ANRS COPANA** cohort, ethical approval was granted by the Ile de France III CPPRB (Paris-Cochin) on 08 August 2003. For the **ANRS CO18 cohort**, ethical approval was granted by the Ile de France VII CPP (Bicêtre) on 11 May 2005 and by DGS (Direction Générale de la Santé) on 25 June 2005. For the **Cascade Study**, ethical approval for collaborating cohorts was granted by the following committees: Austrian HIV Cohort Study: Ethik-Kommission der Medizinischen Universität Wien, Medizinische Universität Graz–Ethikkommission, Ethikkommission der Medizinischen Universität Innsbruck, Ethikkommission des Landes Oberösterreich, Ethikkommission für das Bundesland Salzburg; PHAEDRA cohort: St Vincent's Hospital Human Research Ethics Committee; Southern Alberta Clinic Cohort: Conjoint Health Research Ethics Board of the Faculties of Medicine, Nursing and Kinesiology, University of Calgary; Aquitaine Cohort: Commission Nationale de l'Informatique et des Libertés (CNIL); French Hospital Database: Commission nationale de l'informatique et des libertés (CNIL); French PRIMO Cohort: Comite Consultatif de Protection des Personnes dans la Recherché Biomedicale (CPPRB); SEROCO Cohort: Commission Nationale de l'Informatique et des Libertés (CNIL); German HIV-1 Seroconverter Study: Charité, University Medicine Berlin; AMACS: Bioethics & Deontology Committee of Athens University Medical School and the National Organization of Medicines; Greek Haemophilia Cohort: Bioethics & Deontology Committee of Athens University Medical School and the National Organization of Medicines; ICoNA cohort: San Paolo Hospital Ethic Committee; Italian Seroconversion Study: Comitato etico dell'Istituto Superiore di Sanità; Amsterdam Cohort Studies in Homosexual Men and IDUs: Academic Medical Centre, University of Amsterdam; Oslo and Ulleval Hospital Cohorts: Regional komite for medisinsk forskningsetikk – Øst- Norge (REK 1); Badalona IDU Hospital Cohort: Comité Ético de Investigación Clínica del Hospital Universitari Germans Trias i Pujol; CoRIS-scv: Comité Ético de Investigación Clínica de La Rioja; Madrid Cohort: Ethics Committee of Universidad Miguel Hernandez de Elche; Valencia IDU Cohort: Comité Etico de Investigación Clínica del Hospital Dr. Peset-Valencia; Swiss HIV Cohort Study: Kantonale Ethikkommission, spezialisierte Unterkommission Innere Medizin, Ethikkommission beider Basel, Kantonale Ethikkommission Bern, Comité départemental d'éthique de médecine et médecine communautaire, Commission d'éthique de la recherche clinique, Université de Lausanne, Comitato etico cantonale, Ethikkommission des Kantons St.Gallen; UK Register of HIV Seroconverters: South Birmigham REC; Early Infection Cohorts: Kenya Medical Research Institute, Kenyatta National Hospital, Uganda Virus Research Institute Science and Ethics Committee, Uganda National Council for Science and Technology, Uganda Virus Research Institute Science and Ethics Committee, Uganda National Council for Science and Technology, University of Zambia Research Ethics Committee, Emory IRB, National Ethics Committee of Rwanda, University of Cape Town Research Ethics Committee, University of Kwazulu Natal Nelson R Mandela School of Medicine; Genital Shedding Study Cohort: University Hospitals of Cleveland, IRB for Human Investigation (CWRU), AIDS Research Committee (ARC), STD/AIDS Control Programme, Uganda Ministry of Health, Committee on Human Research (CHR), Office of Research Administration (UCSF), Biomedical Research & Training Institute (BRTI)–Zimbabwe, Institutional Review Office, Fred Hutchinson Cancer Research Center, Medical Research Council of Zimbabwe (MRCZ). For the **SCOPE study** ethical approval was granted by the Committee on Human Research at the University of California, San Francisco and for the **International HIV Controllers study**: ethical approval was granted by the Partners Human Research Committee at Massachusetts General Hospital, Boston, MA. Written informed consent was obtained from all participants.

**The ANRS HIV Controllers cohort study group:**

Dr Olivier Lambotte, Prof. Jean-François Delfraissy, Service de Médecine Interne, Hôpital de Bicêtre, Le Kremlin Bicêtre. Prof. Daniel Séréni, Service de Médecine Interne, Hôpital Saint Louis, Paris. Dr Isabelle Louis, Prof. Philippe Morlat, Service de Médecine Interne, Hôpital Saint André, Bordeaux. Dr Zucman, Prof. Olivier Blétry, Service de Médecine Interne, Hôpital Foch, Suresnes. Mme Michelle Pauchard, Prof. François Bricaire, Prof. Christine Katlama, Service des Maladies Infectieuses, Hôpital Pitié-Salpêtrière, Paris. Mme Marie-Pierre Pietri,Prof. Dominique Salmon-Céron, Service de Médecine Interne 2, Hôpital Cochin, Paris. Dr Christine Rouger, Prof. Gérard Rémy, Service des Maladies Infectieuses, Hôpital Robert Debré, Reims. Mr Bernard Warde, Mme Patricia Honoré, Prof. Alain Krivitzky, Service de Médecine Interne et Endocrinologie, Hôpital Avicenne, Bobigny. Mr Richier Laurent, Dr Olivier Patey, Service des Maladies Infectieuses et Tropicales, CHI Villeneuve Saint Georges, Villeneuve Saint Georges. Dr Laurence Gérard, Prof. Eric Oksenhendler, Service d'Immuno-pathologie, Hôpital Saint Louis, Paris. Mme Véronique Ronat, Prof. Frédéric Lucht, Service de Maladie Infectieuse, Hôpital Bellevue, Saint Etienne. Mme Huguette Berthe, Dr P. de Truchis, Service de Maladies Infectieuses et Tropicales, Hôpital Raymond Poincarré, Garches. Dr Michel Malet, Pr. Daniel Vittecoq, Service des Maladies Infectieuses, Hôpital Paul Brousse, Villejuif. Dr Camille Fontaine, Dr Laura Iordache, Pr.G Pialoux, Service des Maladies Infectieuses, Hôpital Tenon, Paris. Mme Dominique Bornarel, Prof. François Boué, Service de Médecine Interne, Hôpital Antoine Béclère, Clamart. Dr Pascale Koussignan, Prof. Laurence Weiss, Service d'Immunologie Clinique, HEGP, Paris. Mme Cécile Dumont, Prof. Alain Sobel, Service d'Immunologie Clinique, Hôpital Henri Mondor, Créteil. Dr Philippe Roussard, Prof. Pierre-Marie Girard, Service des Maladies Infectieuses, Médecin généraliste, Paris. Dr Caroline Lascoux, Dr Diane Ponscarme, Prof. Jean-Michel Molina, Service des Maladies Infectieuses, Hôpital Saint Louis, Paris. Dr Sylvie Abel, Dr André Cabié, Service de Maladies Infectieuses et Tropicales, Hôpital Pierre Zobda-Quitman, Fort de France, Martinique. Mme Isabelle Raymond, Prof. Jean-Marie Ragnaud, Service des Maladies Infectieuses B, Hôpital Pellegrin, Bordeaux. Dr François Jeanblanc, Service d'Immunologie, Pavillon P, Hôpital Edouard Herriot, Lyon. Dr Kouadjo Koffi, Prof. Christian Trépo, Service Hépato-gastroentérologie, Hôpital Hôtel Dieu, Lyon. Dr Eric Peyrouse, Prof. Jean-Albert Gastaut, Unité de Recherche Clinique, Hôpital Sainte Marguerite, Marseille. Mr Hervé Hüe, Prof. François Raffi, Service de Médecine Interne, Hôpital de l’Hôtel Dieu, Nantes. Mme Pascale Nau, Prof. Patrick Choutet, Service des Maladies Infectieuses, Hôpital Bretonneau, Tours. Mme Florence Balsarin, Prof. Patrice Massip, Service des Maladies Infectieuses, Hôpital Purpan, Toulouse. Mr Philippe Feret, Prof. Claude Bazin, Service des Maladies Infectieuses, Hôpital de la Côte de Nacre, Caen. Mme Pavel Simona, Prof. Yves Mouton, Service des Maladies Infectieuses, Hôpital Gustave Dron, Tourcoing. Mr Laurent Richier, Dr Michel Chousterman, Service des Médecine Interne, Centre Hospitalier Intercommunal, Créteil. Dr Christophe Michau, Service de Médecine Interne, Centre Hospitalier de Saint Nazaire, Saint Nazaire. Mme Barbara de Dieulevault, Dr Laurent Hocqueloux, Service de Maladies Infectieuses et Tropicales, Hôpital Orléans la Source, Orléans.

**CASCADE Steering Committee**

Julia Del Amo (Chair), Laurence Meyer (Vice Chair), Heiner C. Bucher, Geneviève Chêne, Osamah Hamouda, Deenan Pillay, Maria Prins, Magda Rosinska, Caroline Sabin, Giota Touloumi.

**CASCADE Co-ordinating Center**

Kholoud Porter (Project Leader), Ashley Olson, Kate Coughlin, Sarah Walker, Abdel Babiker.

**CASCADE Clinical Advisory Board**

Heiner C. Bucher, Andrea De Luca, Martin Fisher, Roberto Muga.

**CASCADE Collaborators**

Australia: PHAEDRA cohort (Tony Kelleher, David Cooper, Pat Grey,Robert Finlayson, Mark Bloch) Sydney AIDS Prospective Study and Sydney Primary HIV Infection cohort (Tony Kelleher, Tim Ramacciotti, Linda Gelgor, David Cooper, Don Smith); Austria: Austrian HIV Cohort Study (Robert Zangerle); Canada: South Alberta clinic (John Gill); Estonia: Tartu Ülikool (Irja Lutsar); France: ANRS CO3 Aquitaine cohort (Geneviève Chêne, Francois Dabis, Rodolphe Thiebaut, Bernard Masquelier), ANRS CO4 French Hospital Database (Dominique Costagliola, Marguerite Guiguet), Lyon Primary Infection cohort (Philippe Vanhems), French ANRS CO6 PRIMO cohort (Marie-Laure Chaix, Jade Ghosn), ANRS CO2 SEROCO cohort (Laurence Meyer, Faroudy Boufassa); Germany: German HIV-1 seroconverter cohort (Osamah Hamouda, Claudia Kücherer, Barbara Bartmeyer); Greece: AMACS (Anastasia Antoniadou, Georgios Chrysos, Georgios L. Daikos); Greek Haemophilia cohort (Giota Touloumi, Nikos Pantazis, Olga Katsarou); Italy: Italian Seroconversion Study (Giovanni Rezza, Maria Dorrucci), ICONA cohort (Antonella d’Arminio Monforte, Andrea De Luca.) Netherlands: Amsterdam Cohort Studies among homosexual men and drug users (Maria Prins, Ronald Geskus, Jannie van der Helm, Hanneke Schuitemaker); Norway: Oslo and Ulleval Hospital cohorts (Mette Sannes, Oddbjorn Brubakk, Anne-Marte Bakken Kran); Poland: National Institute of Hygiene (Magdalena Rosinska); Spain: Badalona IDU hospital cohort (Roberto Muga, Jordi Tor), Barcelona IDU Cohort (Patricia Garcia de Olalla, Joan Cayla), CoRIS-scv (Julia del Amo, Santiago Moreno, Susana Monge); Madrid cohort (Julia Del Amo, Jorge del Romero), Valencia IDU cohort (Santiago Pérez-Hoyos); Switzerland: Swiss HIV Cohort Study (Heiner C. Bucher, Martin Rickenbach, Patrick Francioli); Ukraine: Perinatal Prevention of AIDS Initiative (Ruslan Malyuta); United Kingdom: Health Protection Agency (Gary Murphy), Royal Free haemophilia cohort (Caroline Sabin), UK Register of HIV Seroconverters (Kholoud Porter, Anne Johnson, Andrew Phillips, Abdel Babiker), University College London (Deenan Pillay). African cohorts: Genital Shedding Study (US: Charles Morrison; Family Health International, Robert Salata, Case Western Reserve University, Uganda: Roy Mugerwa, Makerere University, Zimbabwe: Tsungai Chipato, University of Zimbabwe); International AIDS Vaccine Initiative (IAVI) Early Infections Cohort (Kenya, Rwanda, South Africa, Uganda, Zambia: Pauli N. Amornkul, IAVI, USA; Jill Gilmour, IAVI, UK; Anatoli Kamali, Uganda Virus Research Institute/Medical Research Council Uganda; Etienne Karita, Projet San Francisco, Rwanda).

**EuroCoord Executive Board**

Heiner Bucher, Basel Institute for Clinical Epidemiology & Biostatistics University Hospital Basel, Switzerland; Fiona Burns, University College London, UK; Geneviève Chêne, University of Bordeaux II, France; Dominique Costagliola, Institut National de la Santé et de la Recherche Médicale, France; Carlo Giaquinto, Fondazione PENTA, Italy; Di Gibb (Scientific Coordinator), Medical Research Council, UK; Jesper Grarup, Københavns Universitet, Denmark; Ole Kirk, Københavns Universitet, Denmark; Jesper Kjaer, Københavns Universitet, Denmark; Laurence Meyer, Institut National de la Santé et de la Recherche Médicale, France; Alex Panteleev, St. Petersburg City AIDS Center, Russian Federation; Andrew Phillips, University College London, UK, Kholoud Porter, Medical Research Council, UK; Peter Reiss, Academic Medical Center, Netherlands; Claire Thorne (Chair), University College London, UK.

**EuroCoord Council of Partners**

Jean-Pierre Aboulker, Institut National de la Santé et de la Recherche Médicale, France; Jan Albert, Karolinska Institute, Sweden; Silvia Asandi, Romanian Angel Appeal Foundation, Romania; Geneviève Chêne, University of Bordeaux II, France; Dominique Costagliola, INSERM, France; Antonella d’Arminio Monforte, ICoNA Foundation, Italy; Stéphane De Wit, St. Pierre University Hospital, Belgium; Frank De Wolf, Stichting HIV Monitoring, Netherlands; Julia Del Amo, Instituto de Salud Carlos III, Spain; José Gatell, Fundació Privada Clínic per a la Recerca Bíomèdica, Spain; Carlo Giaquinto, Fondazione PENTA, Italy; Osamah Hamouda, Robert Koch Institut, Germany; Igor Karpov, University of Minsk, Belarus; Bruno Ledergerber, University of Zurich, Switzerland; Jens Lundgren, Københavns Universitet, Denmark; Ruslan Malyuta, Perinatal Prevention of AIDS Initiative, Ukraine; Claus Møller, Cadpeople A/S, Denmark; Andrew Phillips, University College London, UK; Kholoud Porter, Medical Research Council, United Kingdom; Maria Prins, Academic Medical Center, Netherlands; Aza Rakhmanova, St. Petersburg City AIDS Center, Russian Federation; Jürgen Rockstroh (Chair), University of Bonn, Germany; Magda Rosinska, National Institute of Public Health, National Institute of Hygiene, Poland; Claire Thorne, University College London, UK; Giota Touloumi, National and Kapodistrian University of Athens, Greece; Alain Volny Anne, European AIDS Treatment Group, France.

**EuroCoord External Advisory Board**

David Cooper, University of New South Wales, Australia; Nikos Dedes, Positive Voice, Greece; Kevin Fenton, Centers for Disease Control and Prevention, USA; David Pizzuti, Gilead Sciences, USA; Marco Vitoria, World Health Organisation, Switzerland.

**EuroCoord Secretariat**

Kate Coughlin, MRC Clinical Trials Unit, UK; Silvia Faggion, Fondazione PENTA, Italy; Lorraine Fradette, MRC Clinical Trials Unit; Richard Frost, MRC Regional Center London, UK; Miriam Sabin, Københavns Universitet, Denmark; Christine Schwimmer, University of Bordeaux II, France; Martin Scott, UCL European Research & Development Office, UK.

**The International HIV Controllers Study**

**Leadership*:*** Florencia Pereyra, Bruce Walker, Alicja Piechocka-Trocha, Ildiko Toth,Pamela Richtmyer, Brian Block, Brett Baker, Alissa Rothchild, Jeffrey Lian, Jacqueline Proudfoot, Donna Marie L. Alvino.

**Referral team*:*** Brian Agan, Shanu Agarwal, Richard L. Ahern, Brady L. Allen, Sherly Altidor, Eric L. Altschuler, Sujata Ambardar, Kathryn Anastos, Val Anderson, Ushan Andrady, Diana Antoniskis, David Bangsberg, Daniel Barbaro, William Barrie, J. Bartczak, Simon Barton, Patricia Basden, Nesli Basgoz, Nicholaos C. Bellos, Judith Berger, Nicole F. Bernard, Annette M. Bernard, Stanley J. Bodner, Robert K. Bolan, Emilie T. Boudreaux, James F. Braun, Jon E. Brndjar, J. Brown, Sheldon T. Brown, Jedidiah Burack, Larry M. Bush, Virginia Cafaro, John Campbell, Robert H. Carlson, J. Kevin Carmichael, Kathleen K. Casey, Chris Cavacuiti, Gregory Celestin, Steven T. Chambers, Nancy Chez, Lisa M. Chirch, Paul J. Cimoch, Daniel Cohen, Lillian E. Cohn, Brian Conway, David A. Cooper, Brian Cornelson, David T. Cox, Michael V. Cristofano, George Cuchural Jr., Julie L. Czartoski, Joseph M. Dahman, Jennifer S. Daly, Benjamin T. Davis, Kristine Davis, Sheila M. Davod, Edwin DeJesus, Craig A. Dietz, Eleanor Dunham, Michael E. Dunn, Todd B. Ellerin, Joseph J. Eron, John J.W. Fangman, Helen Ferlazzo, Sarah Fidler, Anita Fleenor-Ford, Renee Frankel, Kenneth A. Freedberg, Neel K. French, Jonathan D. Fuchs, Jon D. Fuller, Jonna Gaberman, Joel E. Gallant, Rajesh T. Gandhi, Efrain Garcia, Donald Garmon, Joseph C. Gathe Jr, Cyril R. Gaultier, Wondwoosen Gebre, Frank D. Gilman, Ian Gilson, Paul A. Goepfert, Michael S. Gottlieb, Claudia Goulston, Richard K. Groger, T. Douglas Gurley, Stuart Haber, Robin Hardwicke, W. David Hardy, P. Richard Harrigan, Trevor N. Hawkins, Sonya Heath, Frederick M. Hecht, W. Keith Henry, Melissa Hladek, Robert P. Hoffman, James M. Horton, Ricky K. Hsu, Gregory D. Huhn, Mark L. Illeman, Hans Jaeger, Robert M. Jellinger, Mina John, Jennifer A. Johnson, Kristin L. Johnson, Heather Johnson, Kay Johnson, Jennifer Joly, Wilbert C. Jordan, Carol A. Kauffman, Homayoon Khanlou, Arthur Y. Kim, David D. Kim, Clifford A. Kinder, Laura Kogelman, Erna Milunka Kojic, P. Todd Korthuis, Wayne Kurisu, Douglas S. Kwon, Melissa LaMar, Harry Lampiris, Michael M. Lederman, David M. Lee, Marah J. Lee, Edward T.Y. Lee, Janice Lemoine, Jay A. Levy, Josep M. Llibre, Michael A. Liguori, Susan J. Little, Anne Y. Liu, Alvaro J. Lopez, Mono R. Loutfy, Dawn Loy, Debbie Y. Mohammed, Alan Man, Michael K. Mansour, Vincent C. Marconi, Martin Markowitz, Harold L. Martin Jr., Kenneth Hugh Mayer, M. Juliana McElrath, Theresa A. McGhee, Barbara H. McGovern, Katherine McGowan, Dawn McIntyre, Gavin X. McLeod, Prema Menezes, Greg Mesa, Craig E. Metroka, Dirk Meyer-Olson, Andy O. Miller, Kate Montgomery, Karam C. Mounzer, Iris Nagin, Ronald G. Nahass, Craig Nielsen, David L. Norene, David H. O'Connor, Jason Okulicz, Edward C. Oldfield III, Susan A. Olender, Mario Ostrowski, William F. Owen Jr., Jeffrey Parsonnet, Andrew M. Pavlatos, Alicja Piechocka-Trocha, Aaron M. Perlmutter, Jonathan M. Pincus, Leandro Pisani, Lawrence Jay Price, Laurie Proia, Richard C. Prokesch, Heather Calderon Pujet, Moti Ramgopal, Michael Rausch, J. Ravishankar, Frank S. Rhame, Constance Shamuyarira Richards, Douglas D. Richman, Gregory K. Robbins, Berta Rodes, Milagros Rodriguez, Richard C. Rose III, Eric S. Rosenberg, Daniel Rosenthal, Polly E. Ross, David S. Rubin, Elease Rumbaugh, Luis Saenz, Michelle R. Salvaggio, William C. Sanchez, Veeraf M. Sanjana, Steven Santiago, Wolfgang Schmidt, Hanneke Schuitemaker, Philip M. Sestak, Peter Shalit, William Shay, Vivian N. Shirvani, Vanessa I. Silebi, James M. Sizemore Jr., Paul R. Skolnik, Marcia Sokol-Anderson, James M. Sosman, Paul Stabile, Jack T. Stapleton, Francine Stein, Hans-Jurgen Stellbrink, F. Lisa Sterman, Valerie E. Stone, David R. Stone, Giuseppe Tambussi, Randy A. Taplitz, Ellen M. Tedaldi, Amalio Telenti, Richard Torres, Lorraine Tosiello, Cecile Tremblay, Marc A. Tribble, Phuong D. Trinh, Anthony Vaccaro, Emilia Valadas, Thanes J. Vanig, Isabel Vecino, Wenoah Veikley, Barbara H. Wade, Charles Walworth, Chingchai Wanidworanun, Douglas J. Ward, Robert D. Weber, Duncan Webster, Steve Weis, David A. Wheeler, David J. White, Ed Wilkins, Alan Winston, Clifford G. Wlodaver, Angelique van 't Wout, David P. Wright, Otto O. Yang, David L. Yurdin, Brandon W. Zabukovic, Kimon C. Zachary, Beth Zeeman, Meng Zhao
